# Disturbed balance in the expression of MMP9 and TIMP3 in cerebral amyloid angiopathy-related intracerebral haemorrhage

**DOI:** 10.1186/s40478-020-00972-z

**Published:** 2020-07-06

**Authors:** Lieke Jäkel, H. Bea Kuiperij, Lara P. Gerding, Emma E. M. Custers, Emma van den Berg, Wilmar M. T. Jolink, Floris H. B. M. Schreuder, Benno Küsters, Catharina J. M. Klijn, Marcel M. Verbeek

**Affiliations:** 1 Department of Laboratory Medicine, Radboud University Medical Center, Nijmegen, The Netherlands; 2 Department of Neurology, Radboud Alzheimer Centre, Radboud University Medical Center, Donders Institute for Brain, Cognition and Behaviour, Nijmegen, The Netherlands; 3 Department of Neurology and Neurosurgery, University Medical Center Utrecht Brain Center, Utrecht University, Utrecht, The Netherlands; 4 Department of Pathology, Radboud University Medical Center, Nijmegen, The Netherlands

**Keywords:** Matrix metalloproteinase 9, Tissue inhibitor of metalloproteinases 3, Cerebral amyloid angiopathy, Intracerebral haemorrhage, Alzheimer’s disease, Amyloid β protein

## Abstract

Cerebral amyloid angiopathy (CAA) is characterized by the deposition of the amyloid β (Aβ) protein in the cerebral vasculature and poses a major risk factor for the development of intracerebral haemorrhages (ICH). However, only a minority of patients with CAA develops ICH (CAA-ICH), and to date it is unclear which mechanisms determine why some patients with CAA are more susceptible to haemorrhage than others. We hypothesized that an imbalance between matrix metalloproteinases (MMPs) and their inhibitors (TIMPs) contributes to vessel wall weakening. MMP9 plays a role in the degradation of various components of the extracellular matrix as well as of Aβ and increased MMP9 expression has been previously associated with CAA. TIMP3 is an inhibitor of MMP9 and increased TIMP3 expression in cerebral vessels has also been associated with CAA. In this study, we investigated the expression of MMP9 and TIMP3 in occipital brain tissue of CAA-ICH cases (*n* = 11) by immunohistochemistry and compared this to the expression in brain tissue of CAA cases without ICH (CAA-non-haemorrhagic, CAA-NH, *n* = 18). We showed that MMP9 expression is increased in CAA-ICH cases compared to CAA-NH cases. Furthermore, we showed that TIMP3 expression is increased in CAA cases compared to controls without CAA, and that TIMP3 expression is reduced in a subset of CAA-ICH cases compared to CAA-NH cases. In conclusion, in patients with CAA, a disbalance in cerebrovascular MMP9 and TIMP3 expression is associated with CAA-related ICH.

## Introduction

Cerebral amyloid angiopathy (CAA) is characterized by the accumulation of the amyloid-β (Aβ) protein in cerebral blood vessel walls. CAA most commonly occurs in small- to medium-sized arteries in the cortex and leptomeninges [50]. The occurrence of CAA in leptomeningeal vessels seems to precede involvement of cortical vessels [1, 38] and is has been suggested that the occipital lobe is most severely affected [1, 2, 17, 43, 44, 51]. CAA can be found in 70 to 100% of the patients with Alzheimer’s disease (AD) [3, 10, 23, 54], but also occurs in 35 to 50% of the healthy elderly population [3, 25, 43, 45]. CAA has been strongly associated with the occurrence of lobar intracerebral haemorrhages (ICH) [26, 32, 36, 37]. In addition to the development of ICH, consequences of CAA may include an increased risk of developing lobar intracerebral microhaemorrhages and cognitive impairment [5, 47]. Furthermore, CAA is associated with compromised blood-brain barrier (BBB) integrity, weakened vessel walls, and leaky microvasculature [11, 12, 21].

The molecular mechanisms leading to rupture of CAA-affected vessels remain largely unclear. Matrix metalloproteinases (MMPs) have been suggested to play a role in CAA and CAA-related ICH. MMPs are a family of zinc-dependent endopeptidases that play a role in the degradation of the extracellular matrix (ECM) [22, 29, 31]. MMP9, also known as gelatinase B, degrades ECM components, including several types of collagen, fibronectin, and laminin [49]. MMP9 immunoreactivity has been observed in amyloid-laden vessels in both mouse models of CAA and human AD brain tissue [13, 19, 55]. Isolated rat brain microvessels that were exposed to Aβ40 had elevated expression of MMP9 [13]. In post-mortem brain tissue of human CAA cases with or without concomitant AD pathology, MMP9 expression was associated with Aβ, whereas no MMP9 immunoreactivity has been observed in control cases [55]. In addition, MMP9 has been widely associated with ICH and BBB disruption in both humans and animal studies [12, 28, 33–35, 46].

Tissue inhibitors of metalloproteinases (TIMPs) regulate the activity of MMPs and may therefore be indirectly involved in the regulation of ECM degradation and thus the development of haemorrhages. TIMP3 can form a stable complex with pro-MMP9 [29], and has been shown to inhibit MMP9 activity [8]. Using immunohistochemistry (IHC), next to other techniques, increased cerebrovascular TIMP3 accumulation in brain vessels has been detected in patients with CAA [24], and patients with cerebral autosomal dominant arteriopathy with subcortical infarcts and leukoencephalopathy (CADASIL) [27], a hereditary form of cerebral small vessel disease.

Despite that MMP9 and TIMP3 have been associated with CAA and CAA-related ICH in the past, it is unclear why the cerebrovascular accumulation of Aβ results in ICH in some, but not in all patients. We hypothesized that an imbalance between MMPs and TIMPs contributes to vessel wall weakening and subsequent haemorrhage. We investigated the expression of MMP9 and TIMP3 in brain tissue of CAA cases that developed ICH (CAA-ICH), and compared it to the expression in brain tissue of CAA cases without ICH (CAA-non-haemorrhagic, CAA-NH).

## Methods

### Human brain tissue

*Post-mortem* brain tissue was obtained from Radboudumc Nijmegen, the University Medical Center Utrecht (UMCU), and the Netherlands Brain Bank (NBB), and included 18 CAA-NH and 11 CAA-ICH cases. Groups were age- and sex matched (Table 1). CAA-NH and CAA-ICH cases were selected based on the presence of moderate to severe CAA according to neuropathological assessments in routine autopsy reports. All CAA-ICH cases had experienced lobar ICH, confirmed by neuropathological assessment (Table 1, Additional files 1 and 2). For comparison of TIMP3 protein expression, we included 11 controls without CAA and without ICH, obtained from the Radboudumc Nijmegen, selected based on the absence of neurological disorders and amyloid pathology according to clinical records and autopsy reports (27% female, mean age 74.4 ± 6.6 years, age- and sex-matched with CAA-NH and CAA-ICH groups). Blocks of cortical tissue from the occipital lobe of patients and controls were fixed and embedded in paraffin. We assessed occipital lobe tissue, as this brain region is generally most severely affected by CAA [1]. Tissue was sliced into 4 μm thick sections and mounted on New Silane micro slides for subsequent immunohistochemical (IHC) analysis. Brain samples obtained from the NBB, Netherlands lnstitute for Neuroscience, Amsterdam (open access: www.brainbank.nl), had been collected from donors that had provided written informed consent for the use of autopsy material and clinical information for research purposes. The study was performed in accordance with local regulations and approved by the medical research ethics committee of the UMCU (reference number 17–092). The use of autopsy materials from the Radboudumc was approved by the local ethics committee (reference number 2015–2215). Samples were used anonymously in accordance with the Code of Conduct of the Federation of Medical Scientific Societies in The Netherlands.

**Table 1:** Study group characteristics

	CAA-NH	CAA-ICH	*P*-value
N	18	11	
Age (mean ± sd)	72.9 (12.4)	76.6 (4.7)	0.27 ^a^
Sex (% female)	39	36	1.00 ^b^
CAA grade (mean ± sd) *	3.1 (0.8)	3.5 (0.5)	0.14 ^b^
Demented	Dementia = 12 No dementia = 3 Not reported = 2 Down Syndrome = 1	Dementia = 5 No dementia = 4 Not reported = 2	0.37 ^c^
Location ICH	N.A.	Frontal; n = 2 Fronto-parietal; *n* = 2 Fronto-temporal; *n* = 1 Temporal; *n* = 1 Temporo-parietal; n = 1 Parieto-occipital; *n* = 3 Not reported = 1	N.A.

Abbreviations: *CAA-NH* CAA non-haemorrhagic, *CAA-ICH* CAA-related ICH, *N.A.* not applicable. * CAA grading according to Olichney et al., [30]. ^a^Assessed by t-test; ^b^Assessed by Fishers exact test; ^c^Assessed by chi-square test. See Additional files 1 and 2 for detailed pathological information reported per case

### Immunohistochemistry

Of every case, one occipital lobe section was stained for Aβ, MMP9, and TIMP3 each. Sections were deparaffinized in xylene, rinsed in ethanol, and washed with demi water, before washing in TBS (for MMP9 IHC), TBS supplemented with 0.025% triton (TBS-T; for TIMP3 IHC), or PBS supplemented with 0.1% Tween-20 (PBS-T; for Aβ IHC). Aβ antigen retrieval was achieved by 20 min incubation with neat formic acid. Heat-induced antigen retrieval of MMP9 and TIMP3 was performed by boiling in citrate buffer for 10 min. In addition, TIMP3 sections were incubated with proteinase K (Qiagen, Hilden, Germany, cat: 19133, diluted 1:50 in TBS) for 5 min. Sections were washed and subsequently treated with 3% H_2_O_2_ in methanol for 15 min at room temperature (RT) to block endogenous peroxidase activity, before washing and 30 min incubation with 5% normal goat (TIMP3 IHC) or horse (MMP9 and Aβ IHC) serum diluted in 1% BSA-PBS (PBS supplemented with 1% bovine serum albumin) to block non-specific antibody binding. This was followed by another washing step, before sections were incubated overnight at 4 °C with rabbit-anti-TIMP3 (Abcam, Cambridge, UK, cat: Ab93637, diluted 1:1600 in PBST) or mouse-anti-MMP9 (Invitrogen, Waltham, MA, cat: MA5–14228, diluted 1:50 in 3% BSA-PBS), or 90 min at RT with mouse-anti-Aβ (4G8, Biolegend, San Diego, CA, cat; 800,701, diluted 1:4000 in PBS). Then, sections were washed and incubated 30 min at RT with biotinylated goat-anti-rabbit (Vector Laboratories, Burlingame, CA, cat: BA-2000), or horse-anti-mouse (Vector Laboratories, cat: BA-2000), diluted 1:200 in 1% BSA-PBS. After another washing step, sections were incubated 30 min at RT with Avidin-Biotin complex (Vector Laboratories, cat: PK-4000, diluted 1:100 in 1% BSA-PBS). Subsequently, sections were washed, incubated with diaminobenzidine for 7 min and counterstained with haematoxylin. Finally, sections were rinsed with ethanol, washed with xylene and mounted with Quick D mounting medium. Appropriate negative controls, including isotype controls and secondary antibody incubation after omitting the primary antibody, were included.

### Quantification

#### CAA grading

CAA burden was determined according to the previously described method of Olichney et al. [30] by two independent raters (EEMC and LPG), who were blinded to clinical diagnosis and had excellent interrater agreement (kappa 0.87). A third researcher was consulted in case of disagreement. In brief, tissue was scored with a severity ranging from zero to four. A score of zero indicated that neither leptomeningeal nor superficial cortical vessels were stained for Aβ. A score of one corresponded to scattered staining of either leptomeningeal or cortical blood vessels. A score of two meant that at least a few vessels in the leptomeninges or neocortex were circumferentially stained for Aβ. A score of three reflected a widespread distribution of circumferential Aβ staining in leptomeningeal and cortical vessels. A score of four indicated widespread distribution of circumferential Aβ staining in leptomeningeal and cortical vessels, with additional dysphoric changes [30].

#### Cerebrovascular MMP9 and TIMP3

Sections were scanned at 10x objective magnification and digitized using a 3D Histech P100 scanner (3DHistech, Budapest, Hungary). Quantification was performed by two independent raters (EEMC and LPG), who were blinded to clinical diagnosis. As both MMP9 and TIMP3 staining were largely restricted to large caliber vessels, only vessels with ≥30 μm diameter were included in quantification. The grade of MMP9 and TIMP3 staining in CAA-NH and CAA-ICH cases was scored in all leptomeningeal vessels in the section as follows: Full (> 90% of a vessels circumference and thickness stained) and partial (between 10 and 90% of a vessels circumference and thickness stained). Data was expressed as percentage of vessels with full and partial staining, to normalize for differences in the numbers of leptomeningeal vessels between cases. In the cortex, full (i.e. > 90%) and partial (i.e. 10–90%) staining was scored in all cortical vessels, and data was expressed as number of fully and partially stained vessels per cm^2^, to normalize for differences in cortical area between cases. For some analyses, data of full and partial staining in vessels was combined (“stained”), whereas for other analyses, numbers of fully and partially stained vessels were analyzed separately.

MMP9 and TIMP3 staining was also assessed in unaffected vessels of the investigated tissue (“internal controls”); for this we assessed MMP9 and TIMP3 staining in a minimum of 10 and a maximum of 25 Aβ-negative leptomeningeal and cortical vessels (≥30 μm diameter) of each CAA case (including both CAA-NH and CAA-ICH cases). Data was expressed as percentage of fully and partially MMP9- or TIMP3-stained vessels. As only cases with advanced stages of CAA were included in our study, several sections contained fewer than 10 Aβ-negative vessels; these cases were excluded from further analysis of the internal controls. As, in contrast to MMP9, TIMP3 has only recently for the first time been associated with CAA [24], TIMP3 staining was in addition assessed in brain tissue from 11 patients free of neurological disease (“external controls”), identical to our assessment of CAA-NH and CAA-ICH cases. TIMP3 staining in the control cases was compared to the staining of the CAA cohort.

#### Colocalization of MMP9 and TIMP3 with Aβ

To assess whether findings were dependent on the presence of Aβ in cerebral vessels, we assessed the staining of MMP9 and TIMP3 in a subset of Aβ-stained vessels in CAA-NH vs. CAA-ICH cases. For this purpose, serial sections stained for Aβ and MMP9 or TIMP3 were studied. TIMP3 and MMP9 staining was scored (as full or partial) in a minimum of 10 and a maximum of 25 fully Aβ-stained (i.e. > 90% of a vessel’s circumference and thickness was stained for Aβ) vessels (≥30 μm diameter) per case. Data was expressed as percentage of fully or partially MMP9- or TIMP3-stained vessels. Leptomeningeal and cortical vessels were assessed separately. MMP9 and TIMP3 staining of amyloid plaques was globally evaluated as well.

### Statistical analysis

Statistical analysis was performed using Graphad Prism 5 (La Jolla, CA, USA) and IBM SPSS Statistics v. 25.0 (Armonk, NY, USA). Groups were compared using multiple linear regression analysis with age and sex as covariates. In case of non-normal distribution of residuals, square root or logarithmic transformations were applied. Correlations between CAA grade and protein expression, and between TIMP3 and MMP9 staining, were assessed using Spearman’s test. The threshold for statistical significance was set at 5%.

## Results

### Cerebrovascular MMP9 expression in CAA-NH and CAA-ICH cases

**Fig. 1:**
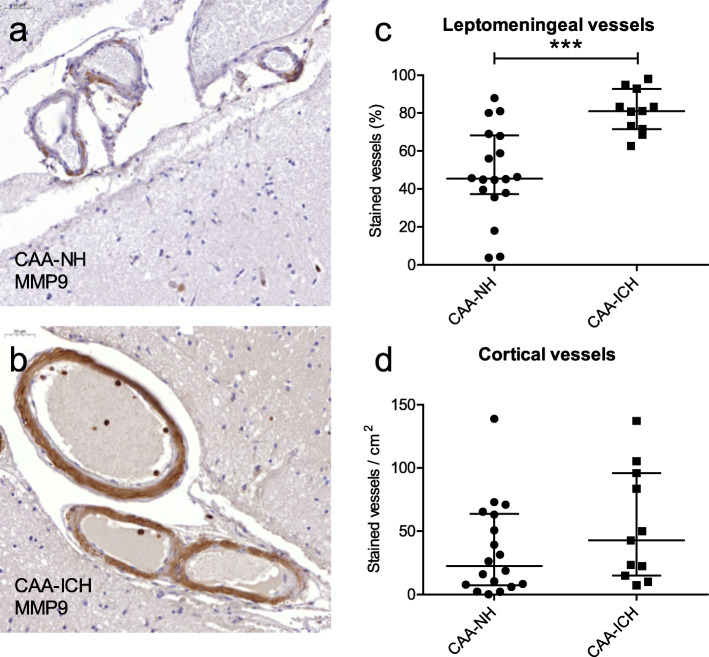
Cerebrovascular MMP9 expression in CAA-NH and CAA-ICH cases. Representative examples of MMP9 staining in a CAA-NH case (**a**), and a CAA-ICH case (**b**), representing the median values as shown in c. Leptomeningeal vessels are stained in both cases: in CAA-NH many vessels are only partially stained, whereas in CAA-ICH cases many vessels are fully stained. Overall, CAA-ICH cases had a significantly higher percentage of MMP9-stained leptomeningeal vessels (either fully or partially stained) compared to CAA-NH cases (**c**). The numbers of MMP9-stained cortical vessels did not differ between groups (**d**). Plots indicate median values with interquartile range. CAA-NH = CAA-non haemorrhagic, CAA-ICH = CAA-related ICH. ***p ≤ 0.001

The percentage of leptomeningeal vessels that was stained for MMP9 was significantly higher in CAA-ICH (median 81%) compared to CAA-NH cases (median 45%, *p <* 0.0005; Fig. 1a-c). The number of cortical vessels stained for MMP9 was not different between CAA-ICH (median 43/cm^2^) and CAA-NH (median 22/cm^2^, *p* = 0.20; Fig. 1d). Aβ-negative cortical vessels of CAA cases (“internal controls”) showed only minimal MMP9-immunoreactity: 10% of Aβ-negative leptomeningeal vessels and 8% of the Aβ-negative cortical vessels were partially stained for MMP9, whereas none of the vessels were fully stained.

### Full and partial MMP9 staining in CAA-NH and CAA-ICH cases

**Fig. 2:**
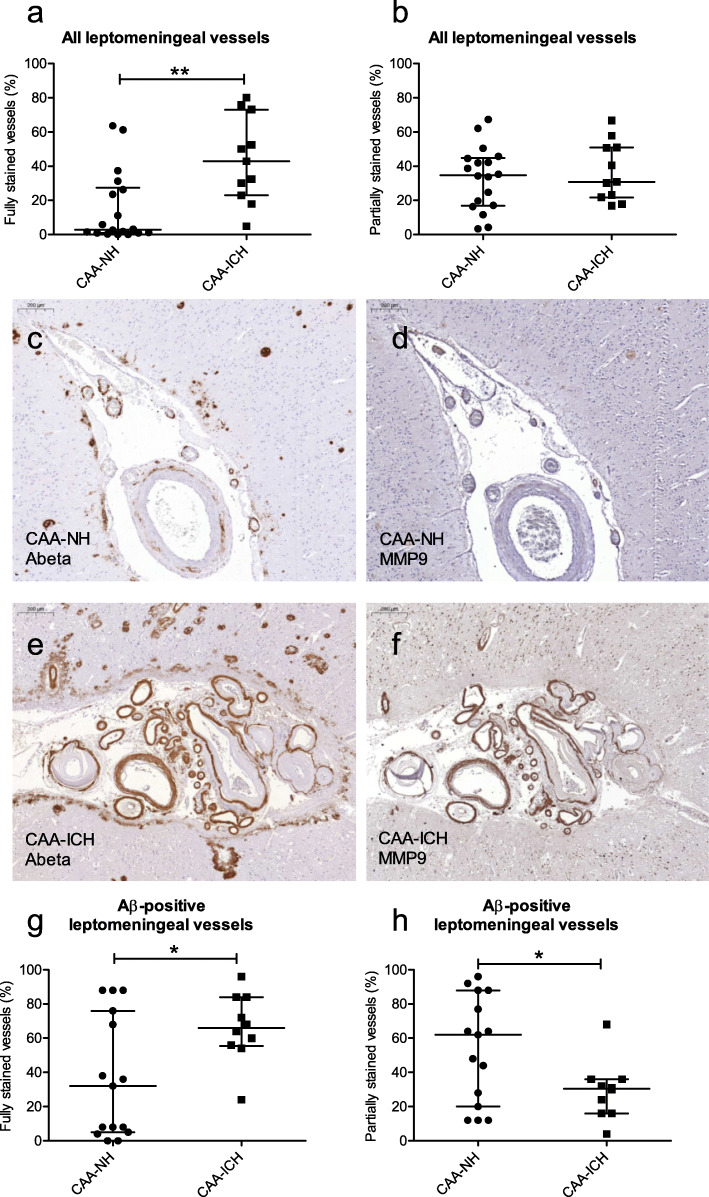
Quantification of MMP9-stained vessels in CAA-NH and CAA-ICH cases, classified according to staining grade. The percentage of leptomeningeal vessels with full (**a**), but not partial (**b**) MMP9 staining was higher in CAA-ICH cases compared to CAA-NH cases. Serial sections of a representative CAA-NH (**c**, **d**) and a representative CAA-ICH (**e**, **f**) case stained for Aβ (**c**, **e**) and MMP9 (**d**, **f**). Scale bar = 200 μm. Compared to CAA-NH cases, CAA-ICH cases had more fully Aβ-stained leptomeningeal vessels that were also fully stained for MMP9 (**g**), but fewer Aβ-stained vessels that had partial MMP9 staining (**h**). Plots indicate median values with interquartile range. CAA-NH = CAA-non haemorrhagic, CAA-ICH = CAA-related ICH. *p ≤ 0.05; **p ≤ 0.01

We separately assessed and compared fully and partially MMP9-stained vessels between CAA-ICH and CAA-NH cases. A significantly higher percentage of fully (median 43% vs. 3%, *p* = 0.001; Fig. 2a), but not of partially (31% vs. 35%, *p* = 0.67; Fig. 2b) MMP9-stained leptomeningeal vessels was observed in CAA-ICH compared to CAA-NH cases, suggesting that the higher number of MMP9-stained vessels in CAA-ICH was mainly driven by a higher number of fully stained vessels. We did not observe significant differences in the numbers of cortical vessels with full (median 23 vs. 4 vessels/cm^2^, *p* = 0.09) or partial (median 16 vs. 7 vessels/cm^2^, *p* = 0.68) MMP9 staining between CAA-ICH and CAA-NH cases (Additional file 3a, b).

### Colocalization of MMP9 with Aβ

We observed a significant correlation between CAA grade and the percentage of leptomeningeal vessels (*p* = 0.0005, r_s_ = 0.61; Fig. 5a) and numbers of cortical vessels (*p* = 0.0007, r_s_ = 0.59; Fig. 5b) fully stained for MMP9 in the combined CAA cohort (including both NH and ICH cases). Furthermore, assessment of serial sections revealed frequent colocalization of Aβ and MMP9 in leptomeningeal and in cortical vessels (examples in Fig. 2c-f). Of note, we did not observe MMP9 expression in parenchymal Aβ plaques. Since MMP9 expression correlated with CAA grade, we aimed to assess whether the increase of MMP9 expression in CAA-ICH cases was independent of Aβ. For this purpose, we assessed if the higher percentage of fully MMP9-stained leptomeningeal vessels that we observed in CAA-ICH cases could be confirmed if we only evaluated fully Aβ-stained vessels in CAA-ICH vs. CAA-NH cases. The percentage of Aβ-stained leptomeningeal vessels that was also fully stained for MMP9 was indeed higher in CAA-ICH than in CAA-NH cases (median 66% vs. 32%, *p* = 0.013; Fig. 2g). Conversely, the percentage of Aβ-stained leptomeningeal vessels that was only partially stained for MMP9 was smaller in CAA-ICH than CAA-NH cases (median 31% vs. 62%, *p* = 0.009; Fig. 2h). Assessment of MMP9 staining in Aβ-stained cortical vessels showed no differences in the percentages of fully stained vessels (median 61 vs. 60%, *p* = 0.08) or partially stained vessels (median 36 vs. 26%, *p* = 0.87; Additional file 3c, d) between CAA-ICH and CAA-NH cases.

### Cerebrovascular TIMP3 expression in CAA-NH and CAA-ICH cases

**Fig. 3:**
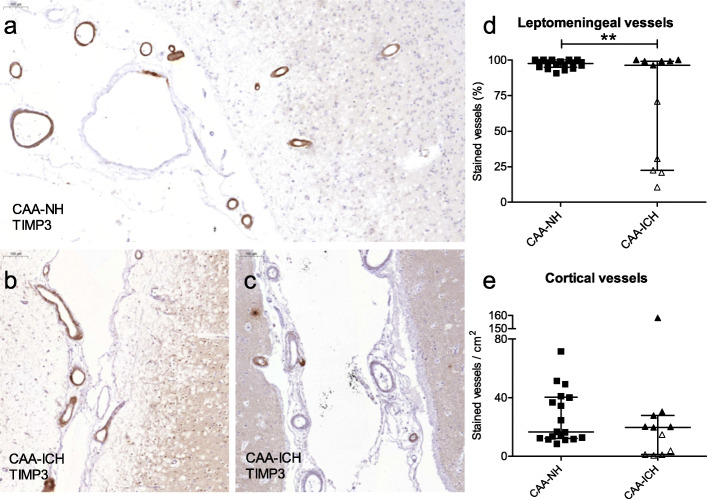
Cerebrovascular TIMP3 expression in CAA-NH and CAA-ICH cases. Representative example of TIMP3 staining in a CAA-NH case (**a**), a CAA-ICH case with high TIMP3 expression (**b**), and a CAA-ICH case with many vessels negative for TIMP3 and only few TIMP3- positive vessels (**c**). Scale bar = 100 μm (**a**). The percentage of TIMP3-stained (full or partial) leptomeningeal vessels was lower in CAA-ICH cases compared to CAA-NH cases, an effect driven by a subgroup of CAA-ICH cases with a substantially lower percentage of TIMP3-stained vessels (open triangles) (**b**). The numbers of cortical vessels stained per cm^2^ did not differ between CAA-ICH cases and CAA-NH cases, although the cases with low TIMP3 expression in leptomeningeal vessels had also low expression in cortical vessels (open triangles) (**c**). Plots indicate median values with interquartile range. CAA-NH = CAA-non haemorrhagic, CAA-ICH = CAA-related ICH. **p ≤ 0.01

The percentage of TIMP3-stained leptomeningeal vessels differed between CAA-ICH cases (median 96%) and CAA-NH cases (median 97%, *p* = 0.003; Fig. 3a-d), a difference driven by a subgroup of CAA-ICH cases with a substantially lower percentage of TIMP3-stained leptomeningeal vessels. The CAA-ICH cases with low TIMP3 expression in leptomeningeal vessels also had low TIMP3 expression in cortical vessels, although the numbers of TIMP3-stained cortical vessels per cm^2^ did not differ between CAA-ICH and CAA-NH cases (20 vs. 17, *p* = 0.61; Fig. 3e.).

### Full and partial TIMP3 staining in CAA-NH and CAA-ICH cases

**Fig. 4:**
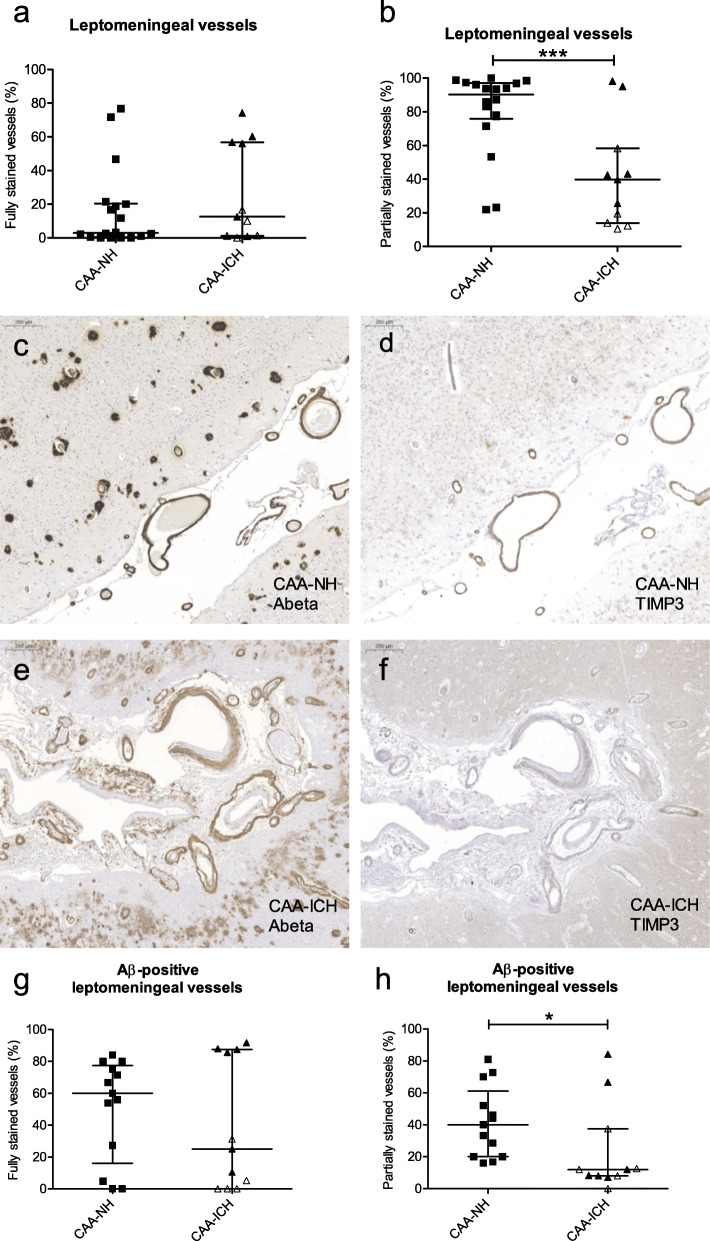
Quantification of TIMP3-stained vessels in CAA-NH and CAA-ICH cases, classified according to staining grade. The percentage of leptomeningeal vessels with full TIMP3 staining did not differ between groups (**a**), whereas the percentage with partial staining was significantly lower in the CAA-ICH group (**b**). Serial sections stained for Aβ (**c**, **e**) and TIMP3 (**d**, **f**) in a CAA-NH case (**c**, **d**) and a CAA-ICH case with low levels of TIMP3 expression (**e**, **f**) showed that these proteins colocalize. Scale bar = 200 μm. Assessment of only fully Aβ-stained leptomeningeal vessels showed no difference in the percentage of vessels that had full TIMP3 staining (**g**), but a lower percentage of partially stained vessels in the CAA-ICH group (**h**). The observed differences were predominantly driven by the subgroup of CAA-ICH cases with low TIMP3 expression (open triangles). Plots indicate median values with interquartile range. CAA-NH = CAA-non haemorrhagic, CAA-ICH = CAA-related ICH, *p ≤ 0.05; ***p ≤ 0.001

We discriminated leptomeningeal vessels that were fully TIMP3-stained from those that had only partial staining. The percentage of leptomeningeal vessels that was fully stained for TIMP3 was similar in CAA-ICH and CAA-NH cases (median 13 vs. 3%, *p* = 0.22; Fig. 4a). In contrast, we observed a significantly lower percentage of partially stained leptomeningeal vessels in CAA-ICH cases compared to CAA-NH cases (median 40 vs. 90 vessels/cm^2^, *p* < 0.0005; Fig. 4b). No differences in the numbers of cortical vessels with full TIMP3 staining (median 1.6 vs. 1.7 vessels/cm^2^, *p* = 0.95) or partial TIMP3 staining (median 11 vs. 13 vessels/cm^2^, *p* = 0.58; Additional file 4a, b) were observed between CAA-ICH and CAA-NH cases.

### Colocalization of TIMP3 with Aβ

**Fig. 5:**
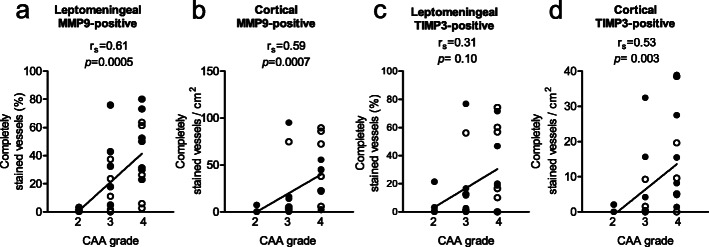
The expression of MMP9 and TIMP3-stained vessels correlated with CAA grade. The percentage of fully MMP9-stained leptomeningeal (**a**) and number of fully MMP9-stained cortical (**b**) vessels showed a positive correlation with CAA grade. The percentage of fully TIMP3-stained leptomeningeal vessels did not significantly correlate to CAA grade (**c**), whereas the numbers of fully TIMP3-stained cortical (**d**) vessels showed a positive correlation with CAA grade. Solid circles = CAA-NH cases; open circles = CAA-ICH cases; r_s_ = Spearman r

No correlation was observed between the percentage of fully TIMP3-stained leptomeningeal vessels and CAA grade (*p* = 0.10, r_s_ = 0.31, Fig. 5c). The numbers of fully TIMP3-stained cortical vessels correlated with CAA grade (*p* = 0.003, r_s_ = 0.53, Fig. 5d). Assessment of serial sections revealed colocalization of Aβ and TIMP3 in both leptomeningeal and cortical vessels (example in Fig. 4c-f). Of note, TIMP3 did not colocalize with parenchymal Aβ in plaques. We assessed whether the decrease of TIMP3 expression in CAA-ICH cases could be confirmed if we only evaluated Aβ-stained vessels in CAA-ICH vs. CAA-NH cases. Analysis of Aβ-stained leptomeningeal vessels that were also stained (fully & partially) for TIMP3 revealed a difference between CAA-ICH and CAA-NH cases (median 92 vs. 100%, *p* = 0.04; Additional file 4c). Separate assessment of vessels that were either fully or partially TIMP3-stained showed that the percentage of Aβ-stained leptomeningeal vessels fully stained for TIMP3 did not significantly differ between CAA-ICH and CAA-NH cases (median 25 vs. 60%; *p* = 0.67; Fig. 4g). However, the numbers of Aβ-stained leptomeningeal vessels with partial TIMP3 staining differed between CAA-ICH and CAA-NH cases (median 12 vs. 40%; *p* = 0.015; Fig. 4h).

### MMP9:TIMP3 ratio and correlation

**Fig. 6:**
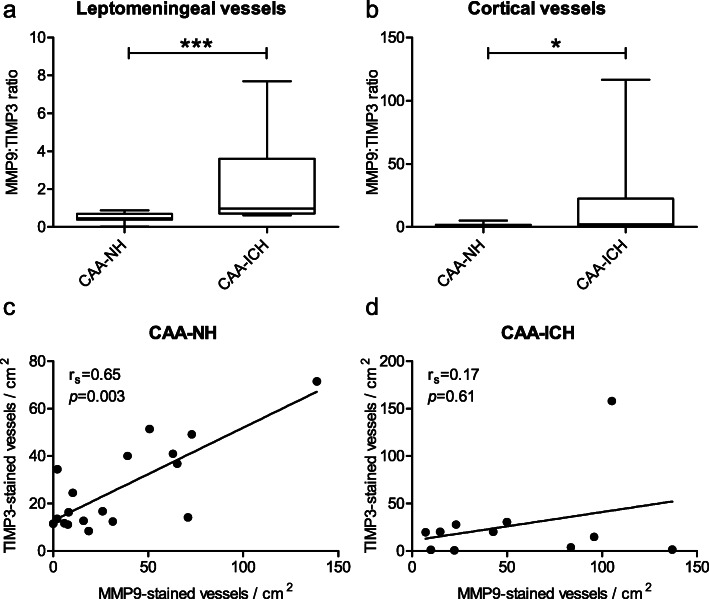
Disturbed balance of MMP9 and TIMP3 in vessels of CAA-ICH cases. The ratio of MMP9-stained (partially or fully) to TIMP3-stained (partially or fully) leptomeningeal vessels (a) and  cortical vessels (b) was higher in CAA-ICH cases compared to CAA-NH casesIn CAA-NH cases, there was a positive correlation between the numbers of cortical vessels stained (partially or fully) for MMP9 and the numbers of cortical vessels stained (partially or fully) for TIMP3 (**c**). Such a correlation was not seen in CAA-ICH cases (**d**). Box plots show median values with the 25th and 75th percentile as boundaries and whiskers indicating minimum and maximum values. CAA-NH = CAA-non haemorrhagic, CAA-ICH = CAA-related ICH, *p ≤ 0.05; ***p ≤ 0.001, r_s_ = Spearman r

The balance between MMP and TIMP activity determines the net ECM degrading potential of MMPs, therefore we assessed the ratio between MMP9 and TIMP3 staining. The ratio between the percentage of MMP9- and TIMP3-stained leptomeningeal vessels was significantly higher in CAA-ICH cases compared to CAA-NH cases (median 1.0 vs. 0.5, *p* = 0.001; Fig. 6a). Likewise, there was a higher MMP9:TIMP3 ratio in cortical vessels of CAA-ICH cases compared to CAA-NH cases (median 2.1 vs. 1.1, *p* = 0.03; Fig. 6b). In CAA-NH cases, there was a positive correlation between the numbers of cortical vessels stained (fully or partially) for MMP9 and the numbers of cortical vessels stained for TIMP3 (*p* = 0.003, r_s_ = 0.65, Fig. 6c). Such a correlation was not seen in CAA-ICH cases (Fig. 6d). These correlation analyses were not performed on data of leptomeningeal vessels, as in most cases all leptomeningeal vessels were (fully or partially) stained for MMP9.

### Cerebrovascular TIMP3 expression in control cases

**Fig. 7:**
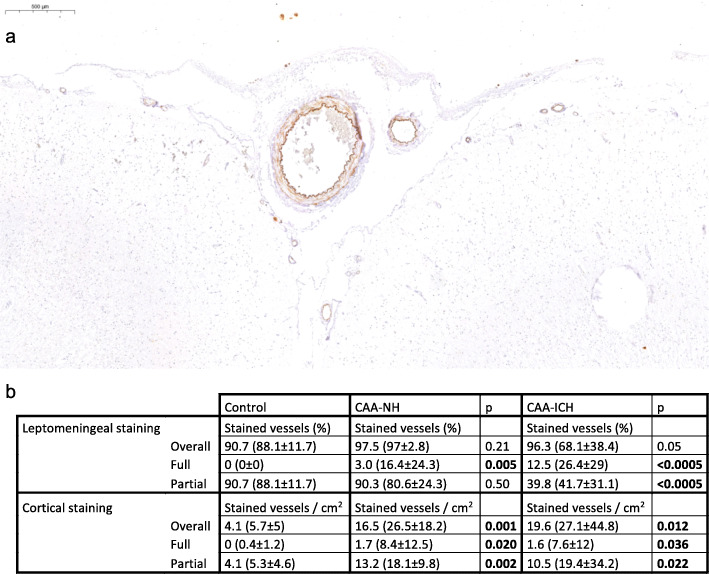
TIMP3 staining in control cases. Representative example of TIMP3 staining in a control case (**a**). Table shows median (mean ± sd) values of the percentages (in case of leptomeningeal) or numbers per cm^2^ (in case of cortical) vessels (**b**). The difference in expression between control cases and CAA-NH or CAA-ICH cases was assessed by linear regression with age and sex as covariates. Indicated in bold are *p*-values < 0.05. CAA-NH = CAA-non haemorrhagic, CAA-ICH = CAA-related ICH

The percentage of TIMP3-stained leptomeningeal vessels did not differ between control cases (example in Fig. 7a) and CAA-NH or CAA-ICH cases. However, the degree of staining was different (Fig. 7b). Control cases had a significantly lower percentage of fully stained leptomeningeal vessels compared to CAA-NH and CAA-ICH cases and a significantly higher percentage of partially TIMP3-stained leptomeningeal vessels compared to CAA-ICH cases. Furthermore, control cases had significantly fewer TIMP3-stained (full or partial) cortical vessels compared to CAA-NH and CAA-ICH cases. These findings are in line with assessment of internal control vessels, which showed that 89% of the Aβ-negative leptomeningeal vessels in CAA cases were stained to some degree for TIMP3, but in most of these vessels (81%) the staining was partial. Of the Aβ-negative cortical vessels, only 5% had (full or partial) TIMP3 staining.

## Discussion

In this study, we show that MMP9 expression is increased in cases with CAA-ICH compared to CAA cases without ICH. Furthermore, we show that there is more extensive TIMP3 staining in CAA cases versus controls and that a subset of CAA-ICH cases has a remarkable loss of TIMP3 expression.

MMP9 has been implicated in the development of ICH and BBB disruption [18, 28, 33–35, 39, 46]. In Tg2576 mice, a model for CAA and AD, microhaemorrhages were associated with MMP9 expression [19]. Administration of recombinant MMP9 to the surface of mouse brains (through craniotomies) resulted in the occurrence of lobar haemorrhages, and Tg2576 mice with severe CAA were more susceptible to this procedure compared to wild-type mice [55]. Human studies revealed increased levels of MMP9 in haemorrhagic areas compared to the contralateral hemisphere of CAA cases that suffered from ICH [14]. Furthermore, Prussian blue positive products, indicative of cerebral microbleeds, were observed in the proximity of a cluster of vessels showing MMP9 immunoreactivity in CAA cases [55]. However, all these findings were either of a qualitative, rather than of a quantitative nature [55], deduced from animal research [19, 55], or focussed on the brain region directly affected by ICH [14].

In our study, we aimed to quantitatively assess the expression of MMP9 in the human cerebrovasculature, without restriction to the immediate proximity of the haemorrhagic site (Table 1). Also, for the first time, we compared cases affected by CAA with and without ICH. We show here that increased vascular MMP9 levels are observed in CAA-ICH cases compared to CAA-NH, driven by a shift from partial MMP9 staining towards full MMP9 staining. Since we studied cerebral vessels distant from the site of ICH, our studies suggest that in CAA-ICH there is a global increased expression of MMP9, which may be mechanistically linked to the ICH. In correspondence with previous findings [55], Aβ-negative cortical vessels of CAA cases showed only minimal MMP9-immunoreactity. We have confirmed previous findings on a correlation between MMP9 staining and CAA grade [55] and on colocalization of MMP9 and Aβ [13, 19], both of which are a logical consequence of the capacity of MMP9 to degrade both soluble and aggregated Aβ [4, 15, 52, 53], and its induced expression in response to Aβ [9, 19].

Our findings on TIMP3 expression are in line with previous publications that reported that TIMP3 expression was more pronounced in CAA patients compared to controls, that TIMP3 colocalized with Aβ in leptomeningeal cerebrovascular arteries [24], and that TIMP3 protein levels were increased in brains of AD patients and a mouse model of AD [16]. In addition, we showed that TIMP3 expression is not restricted to leptomeningeal arteries [24], but also detected in cortical arteries. We also demonstrate for the first time that a subset of CAA-ICH cases has a remarkably low expression level of TIMP3. Again, as we studied cerebral vessels not in the immediate vicinity of the site of ICH, our observations suggest that TIMP3 expression may be globally decreased in (a subset of) CAA-ICH cases, which may be mechanistically linked to the ICH. Not all CAA-ICH cases had decreased TIMP3 expression, indicating that other factors and pathways besides TIMP3 expression influence ICH development.

As TIMP3 has been shown to inhibit MMP9 activity [6, 8], we speculate that TIMP3 expression increases in response to elevated levels of MMP9 and possibly other MMPs in CAA patients, but that this negative feedback mechanism seems to fail in a subset of CAA-ICH cases, resulting in decreased inhibition of MMP9 and therefore increased risk of ICH development. The altered MMP9:TIMP3 ratio that we observed in CAA-ICH cases points towards a disbalance of these proteins. In addition, the positive correlation between the numbers of MMP9- and TIMP3-stained cortical vessels in CAA-NH cases supports the hypothesis of a feedback mechanisms in Aβ-affected vessels. In contrast, the absence of such a correlation in a subset of CAA-ICH cases may indicate that the feedback mechanism is failing in these patients. However, further, mechanistic studies are needed to confirm these hypotheses. Interestingly, increased TIMP3 levels may also contribute to an increased risk of CAA-related ICH, through activation of another pathway, as cellular overexpression of TIMP3 has been shown to redirect amyloid precursor protein (APP) processing towards the amyloidogenic pathway, through inhibition of ADAM10, a metalloproteinase that serves as an α-secretase [16]. By reducing α-cleavage of APP and increasing β-cleavage, Aβ levels may increase as a consequence of TIMP3 overexpression [16]. In the absence of ADAM10 expression data it is not possible to draw conclusions on the potential role of this latter mechanism.

The differential expression patterns of MMP9 and TIMP3 in CAA-ICH cases compared to CAA-NH cases were more pronounced in leptomeningeal vessels compared to cortical vessels. This may, in part, be due to the earlier and more severe accumulation of Aβ in leptomeningeal vessels as compared with cortical vessels [1, 38]. As we showed that both MMP9 and TIMP3 are strongly associated with Aβ, the observed differences in MMP9 and TIMP3 expression may therefore be more pronounced and easier detectable in leptomeningeal vessels. Furthermore, it is possible that altered MMP9 and TIMP3 levels in leptomeningeal vessels may disturb vascular functioning and blood flow and lead to haemorrhages downstream in cortical vessels, which may be in line with a recent observation that vessels do not rupture at the site of Aβ deposition [40, 41], but rather downstream or upstream, possibly mediated by impaired autoregulation. Furthermore, one could speculate that decreased integrity of leptomeningeal vessel walls may contribute to the development of cortical superficial siderosis, which is the deposition of blood-breakdown products in the subarachnoid space and strongly linked to CAA [7, 20].

Small haemorrhagic lesions are frequently observed in CAA patients, and we cannot rule out that such lesions may have influenced results. However, we assessed the presence of small haemorrhagic lesions in the brain tissue by Perls Prussian Blue iron staining. In several CAA-ICH cases, microbleeds were detected (supplementary Table 2). There was no appreciable staining of MMP9 or TIMP3 in the close proximity of microbleeds (Additional file 5), suggesting that such lesions may not or, only to a minor extent, have contributed to the observed differences in MMP9 or TIMP3 expression. Furthermore, even in the absence of (micro) hemorrhages, CAA-affected vessels may be leaky and permeable to plasma proteins [11], potentially resulting in MMP9 upregulation. However, fibrinogen immunostaining, as a proxy of BBB permeability, did not differ between CAA-NH and CAA-ICH cases and did not correlate with MMP9 staining (Additional file 6), making it unlikely that the upregulation of  MMP9 is a result of increased vessel permeability.

Taken together, MMP9 and TIMP3 are directly associated with the presence of CAA. In addition, levels of these proteins are altered in CAA-ICH cases compared to CAA-NH cases. As altered levels of these proteins are directly related to an increased risk of ICH, MMP9 and TIMP3 pathways may have potential as therapeutic targets to prevent ICH in CAA patients. Noteworthy is our observation that MMP9 and TIMP3 are expressed at the site of vascular Aβ accumulation, but not in parenchymal Aβ accumulation (plaques). Of note, although we specifically assessed MMP9 and TIMP3, other members of the MMP and TIMP families may play a role in CAA-related ICH, such as MMP2, which has been previously associated with CAA-related ICH [14].

Our study has several limitations. First, our CAA cohort consists of a heterogenous patient group, with, in addition to moderate to severe CAA, varying degrees of AD pathology. Second, it is possible that not all cases of ICH were only due to CAA, and that other age-related pathological mechanisms were involved. Possibly, different aetiologies of ICH may explain the different TIMP3 expression patterns observed in our CAA-ICH cohort. Furthermore, as tissue of CAA-ICH cases is relatively scarce, we have included tissue from different brain banks, and therefore, we cannot rule out that differences in post-mortem interval and tissue treatment protocols (e.g. formalin exposure) may have affected our results. Finally, the number of cases included in our study was relatively small, and the results of this pilot study need to be validated in a larger cohort.

A strong point of our study is our unique cohort, which enabled us a direct comparison of CAA-ICH cases with CAA-NH cases. Previous observations on increased MMP9 levels in CAA-ICH are based on protein expression in proximity of the haemorrhagic area, compared to expression levels in the contralateral hemisphere [14], which may reflect post-ICH inflammation rather than a pathophysiological mechanism of ICH [35, 42, 48]. Another strong point of our study is the analysis of brain regions distant from the haemorrhagic site. In all cases, we studied the occipital cortex, whereas the haemorrhage usually had occurred in other locations, including the parietal and frontal cortices. Therefore, our data suggest that the observations of increased levels of MMP9 and decreased levels of TIMP3 may be a cause rather than a consequence of ICH, and that post-ICH inflammatory processes possibly only made a minor contribution to the observed differences, as we observed globally changed protein expression levels in the brains of CAA-ICH cases. However, we cannot rule out the possibility that MMP-9 levels in CAA-ICH cases increased post-ICH, especially in case of long time spans between ICH and death. However, previous reports on patients with haemorrhagic stroke did not detect an increase of MMP9-positive vessels in contralateral brain sections, in contrast to perihematomal tissue [13, 35].

In conclusion, we provide evidence that increased cerebrovascular levels of MMP9 and decreased levels of TIMP3 are associated with CAA-related ICH. Future studies are needed to validate these findings in larger data sets, and to determine the mechanistic pathways leading to the altered expression levels of MMP9 and TIMP3.

## Supplementary information

**Additional file 1.** Clinical and pathological information of CAA-NH cases.

**Additional file 2.** Clinical and pathological information of CAA-ICH cases.

**Additional file 3.** Quantification of MMP9-stained cortical vessels in CAA-NH and CAA-ICH cases.

**Additional file 4.** Quantification of TIMP3-stained vessels in CAA-NH and CAA-ICH cases.

**Additional file 5.** Example of a microbleed in a CAA-ICH case.

**Additional file 6.** Cortical fibrinogen staining in CAA-NH and CAA-ICH cases.

## Data Availability

The datasets used and/or analysed during the current study available from the corresponding author on reasonable request.

## Abbreviations

AD: Alzheimer’s disease
Aβ: Amyloid β
APP: Amyloid precursor protein
BBB: Blood-brain barrier
BSA-PBS: PBS supplemented with bovine serum albumin
CAA: Cerebral amyloid angiopathy
CAA-ICH: CAA-related intracerebral haemorrhage
CAA-NH: CAA-non-haemorrhagic
CADASIL: Cerebral autosomal dominant arteriopathy with subcortical infarcts and leukoencephalopathy
ECM: Extracellular matrix
ICH: Intracerebral haemorrhage
IHC: Immunohistochemistry
MMP: Matrix metalloproteinase
NBB: Netherlands Brain Bank
RT: Room temperature
PBS-T: PBS supplemented with 0.1% Tween-20
TBS-T: TBS supplemented with 0.025% Triton
TIMP: Tissue inhibitor of metalloproteinases
UMCU: University Medical Center Utrecht
