# Supplementary material for: Disturbed balance in the expression of MMP9 and TIMP3 in cerebral amyloid angiopathy-related intracerebral haemorrhage
Source: Acta Neuropathol Commun. 2020 Jul 6;8:99. doi: 10.1186/s40478-020-00972-z (PMC7336459; doi:10.1186/s40478-020-00972-z)
Supplement: Supplementary file 1 — Additional file 1. Clinical and pathological information of CAA-NH cases. [file 40478_2020_972_MOESM1_ESM.docx]

**Additional file 1**. Clinical and pathological information of CAA-NH cases

| **No** | **Sex** | **Age** | **Dementia^1^** | **Source tissue** | **Tau pathology (Braak stage)** | **Braak stage; dichotomized^2^** | **Amyloid pathology (CERAD)** | **Post-mortem delay** | **CAA grade^3^** |
| --- | --- | --- | --- | --- | --- | --- | --- | --- | --- |
| 1 | Male | 74 | yes (LBD) | RUMC | 0 | Low | None | <24h | 4 |
| 2 | Female | 83 | no | RUMC | 2 | Low | Sparse | 3d | 4 |
| 3 | Female | 90 | yes (AD) | RUMC | 5 | High | Frequent | nr | 2 |
| 4 | Male | 71 | no | RUMC | nr | Low | nr | 2d | 3 |
| 5 | Male | 73 | yes | RUMC | 2 | Low | Sparse | nr | 3 |
| 6 | Male | 62 | yes (AD) | RUMC | 5 | High | Frequent | 3d | 4 |
| 7 | Male | 55 | no | RUMC | 0 | Low | None | 3d | 3 |
| 8 | Male | 75 | yes (VD) | RUMC | 3 | Low | Sparse | <24h | 3 |
| 9 | Male | 40 | no (Down syndrome) | RUMC | 3 | Low | Sparse | <24h | 2 |
| 10 | Female | 71 | yes (AD) | RUMC | 5 | High | Frequent | 1d | 4 |
| 11 | Female | 74 | yes (AD+VD) | RUMC | 6 | High | Frequent | nr | 3 |
| 12 | Male | 73 | yes (AD+VD) | RUMC | 6 | High | Moderate | 2d | 2 |
| 13 | Female | 76 | yes (AD) | RUMC | 6 | High | Frequent | nr | 2 |
| 14 | Male | 75 | yes (AD) | RUMC | 6 | High | Frequent | <24h | 3 |
| 15 | Female | 87 | yes (AD) | RUMC | 3 | Low | Sparse | 2d | 3 |
| 16 | Male | 77 | nr | RUMC | 2 | Low | Frequent | 1d | 4 |
| 17 | Female | 92 | yes (AD) | RUMC | 4 | High | Sparse | 1d | 2 |
| 18 | Male | 64 | nr | RUMC | 5 | High | Frequent | 1d | 4 |

Legends: ^1^ Suspected type of dementia; ^2^ Low = Braak 0-3, High = Braak 4-6. In some cases, the exact Braak stage was not reported, but the pathology report only mentioned a low degree of tau pathology; ^3^ CAA severity was assessed in an occipital tissue section according to Olichney et al (1996). Abbreviations: AD = Alzheimer’s dementia; LBD = Lewy Body dementia; nr = not reported; RUMC = Radboud university medical center; VD = vascular dementia.
