# Supplementary material for: Disturbed balance in the expression of MMP9 and TIMP3 in cerebral amyloid angiopathy-related intracerebral haemorrhage
Source: Acta Neuropathol Commun. 2020 Jul 6;8:99. doi: 10.1186/s40478-020-00972-z (PMC7336459; doi:10.1186/s40478-020-00972-z)
Supplement: Supplementary file 2 — Additional file 2. Clinical and pathological information of CAA-ICH cases. [file 40478_2020_972_MOESM2_ESM.docx]

**Additional file 2**. Clinical and pathological information of CAA-ICH cases

| **No** | **Sex** | **Age** | **Dementia^1^** | **Source tissue** | **Tau pathology (Braak stage)** | **Braak stage; dichotomized^2^** | **Amyloid pathology (CERAD)** | **Post-mortem delay** | **CAA grade^3^** | **Perls staining^4^** | **Location ICH** | **Studied hemisphere** | **Size ICH** | **Age haemorrhage** |
| --- | --- | --- | --- | --- | --- | --- | --- | --- | --- | --- | --- | --- | --- | --- |
| 1 | Male | 70 | no | RUMC | 0 | Low | None | 6d | 3 | No | Frontal (R); Frontal (L); Temporal (R) | Left | 6x4cm; 4x4cm; 2x1cm | "fairly recent" |
| 2 | Male | 79 | no | RUMC | nr | Low | nr ("some plaques") | nr | 4 | Yes | Temporal (L) | Left | nr | nr |
| 3 | Male | 68 | no | RUMC | nr | Low | nr | 2d | 4 | Yes | Parieto-occipital (L) | Left | nr | 2 days |
| 4 | Male | 75 | yes | RUMC | 4 | High | Moderate | nr | 3 | No | Fronto-parietal (R) | Left | nr | nr |
| 5 | Female | 79 | no | RUMC | nr | Low | nr | nr | 4 | Yes | Parieto-occipital (L) | Left | Diameter ± 5cm | "recent" |
| 6 | Female | 73 | yes (AD) | NBB | 5 | High | Frequent | <24h | 3 | No | Frontal (L) | Left | nr | < 1 day |
| 7 | Female | 83 | yes (AD) | NBB | 4 | High | nr (frequent plaques in hippocampus) | <24h | 4 | Yes | Fronto-parietal (R) | Left | nr | 2 days |
| 8 | Female | 81 | yes (AD) | NBB | 6 | High | Frequent | <24h | 4 | Yes | Frontal (L) | Right | nr | <1 hour |
| 9 | Male | 80 | nr | UMCU | nr | nr | nr | nr | 3 | Yes | nr | nr | nr | nr |
| 10 | Male | 78 | yes | UMCU | 6 | High | Frequent | nr | 3 | No | Temporo-parietal (L+R) | Right | ±50 ml | 5 days |
| 11 | Male | 76 | nr | UMCU | 5 or 6 | High | Frequent | nr | 4 | No | Parieto-occipital (R) | Left | ±40 ml | 2 days |

Legends: ^1^ Suspected type of dementia; ^2^ Low = Braak 0-3, High = Braak 4-6. In some cases, the exact Braak stage was not reported, but the pathology report only mentioned a low degree of tau pathology; ^3^ CAA severity was assessed in an occipital tissue section; ^4^ In occipital sections of these cases, Perls Prussian blue staining was detected, indicative of the presence of microvascular lesions. Abbreviations: AD = Alzheimer’s dementia; LBD = Lewy Body dementia; nr = not reported; NBB = Netherlands Brain bank; RUMC = Radboud university medical center; UMCU = University Medical Center Utrecht; VD = vascular dementia.
