# Supplementary material for: Disturbed balance in the expression of MMP9 and TIMP3 in cerebral amyloid angiopathy-related intracerebral haemorrhage
Source: Acta Neuropathol Commun. 2020 Jul 6;8:99. doi: 10.1186/s40478-020-00972-z (PMC7336459; doi:10.1186/s40478-020-00972-z)
Supplement: Supplementary file 3 — Additional file 3. Quantification of MMP9-stained cortical vessels in CAA-NH and CAA-ICH cases. [file 40478_2020_972_MOESM3_ESM.docx]

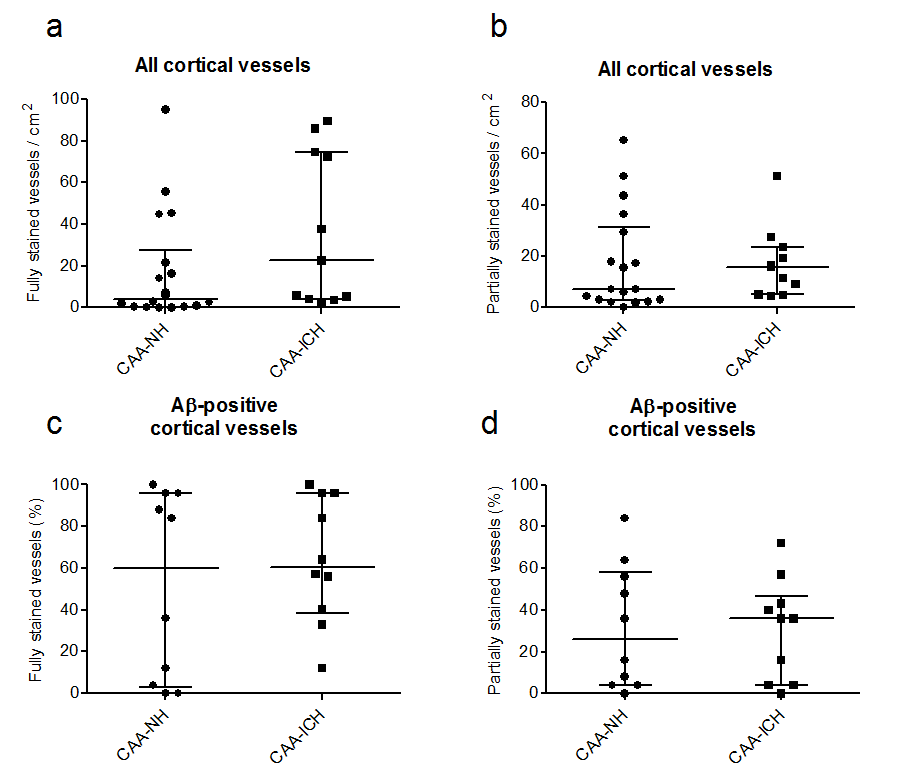


**Additional file 3.** Quantification of MMP9-stained cortical vessels in CAA-NH and CAA-ICH cases, classified according to staining grade (full (a,c) versus partial staining (b,d)). The number of cortical vessels with full (a) and partial (b) MMP9 staining was not different between CAA-ICH and CAA-NH cases. Similarly, the percentage of fully Aβ-stained cortical vessels with full (c) and partial (d) MMP9 staining did not differ between CAA-ICH and CAA-NH cases. CAA-NH = CAA-non haemorrhagic, CAA-ICH = CAA-related ICH.
