# Supplementary material for: Disturbed balance in the expression of MMP9 and TIMP3 in cerebral amyloid angiopathy-related intracerebral haemorrhage
Source: Acta Neuropathol Commun. 2020 Jul 6;8:99. doi: 10.1186/s40478-020-00972-z (PMC7336459; doi:10.1186/s40478-020-00972-z)
Supplement: Supplementary file 4 — Additional file 4. Quantification of TIMP3-stained vessels in CAA-NH and CAA-ICH cases. [file 40478_2020_972_MOESM4_ESM.docx]

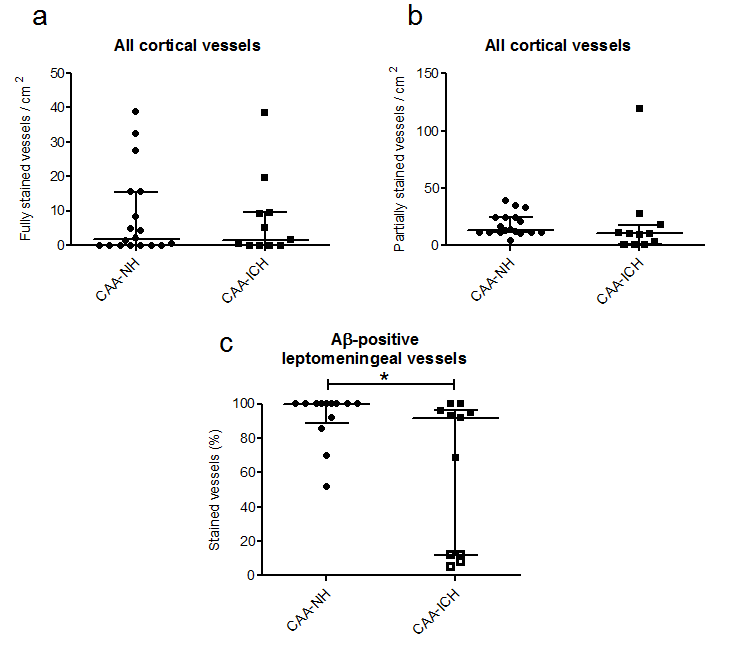


**Additional file 4.** Quantification of TIMP3-stained vessels in CAA-NH and CAA-ICH cases. The number of cortical vessels with full (a) and partial (b) TIMP3 staining was not different between CAA-ICH and CAA-NH cases. However, the percentage of fully Aβ-stained leptomeningeal vessels that was also stained (fully or partially) for TIMP3 (c) was lower in CAA-ICH cases compared to CAA-NH cases. CAA-NH = CAA-non haemorrhagic, CAA-ICH = CAA-related ICH. *p≤0.05.
