# Supplementary material for: Disturbed balance in the expression of MMP9 and TIMP3 in cerebral amyloid angiopathy-related intracerebral haemorrhage
Source: Acta Neuropathol Commun. 2020 Jul 6;8:99. doi: 10.1186/s40478-020-00972-z (PMC7336459; doi:10.1186/s40478-020-00972-z)
Supplement: Supplementary file 5 — Additional file 5. Example of a microbleed in a CAA-ICH case. [file 40478_2020_972_MOESM5_ESM.docx]

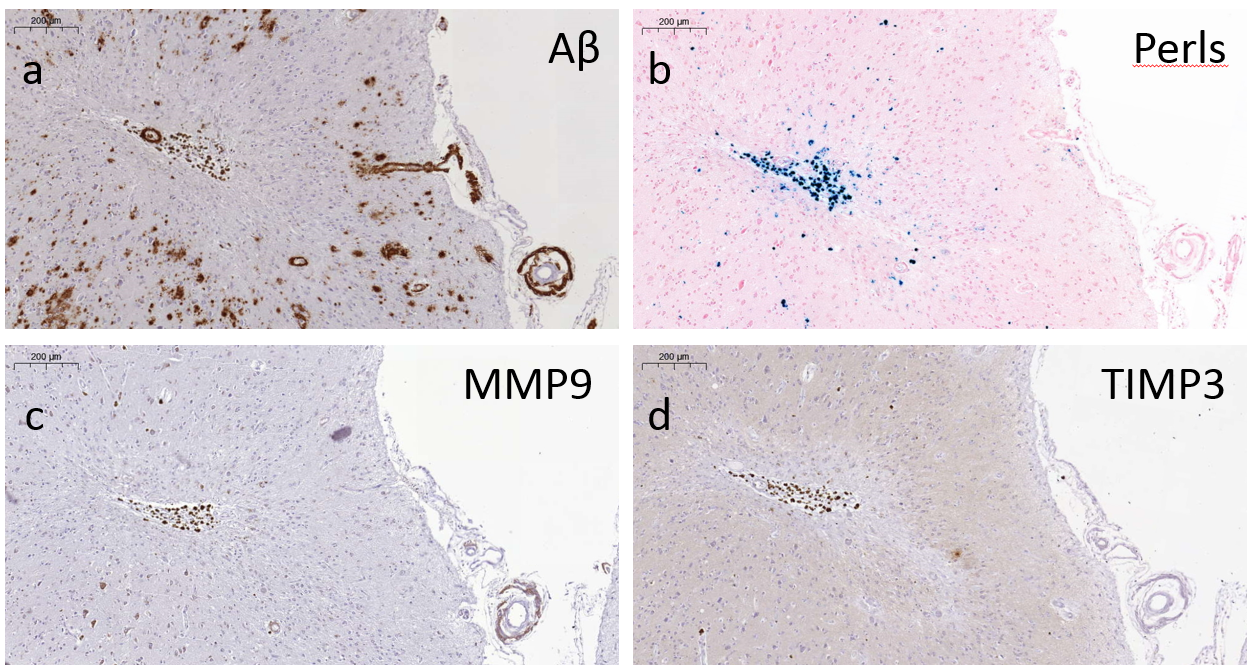


**Additional file 5**. Example of a microbleed in a CAA-ICH case. The perihaemorrhagic area was devoid of Aβ (a), whereas iron particles, indicative of a microbleed, were detected by Perls Prussian blue staining (b). There was no appreciable staining of MMP9 (c) and TIMP3 (d) in the direct proximity of the microbleed. Scale bar = 200 µm.
