# Supplementary material for: Disturbed balance in the expression of MMP9 and TIMP3 in cerebral amyloid angiopathy-related intracerebral haemorrhage
Source: Acta Neuropathol Commun. 2020 Jul 6;8:99. doi: 10.1186/s40478-020-00972-z (PMC7336459; doi:10.1186/s40478-020-00972-z)
Supplement: Supplementary file 6 — Additional file 6. Cortical fibrinogen staining in CAA-NH and CAA-ICH cases. [file 40478_2020_972_MOESM6_ESM.docx]

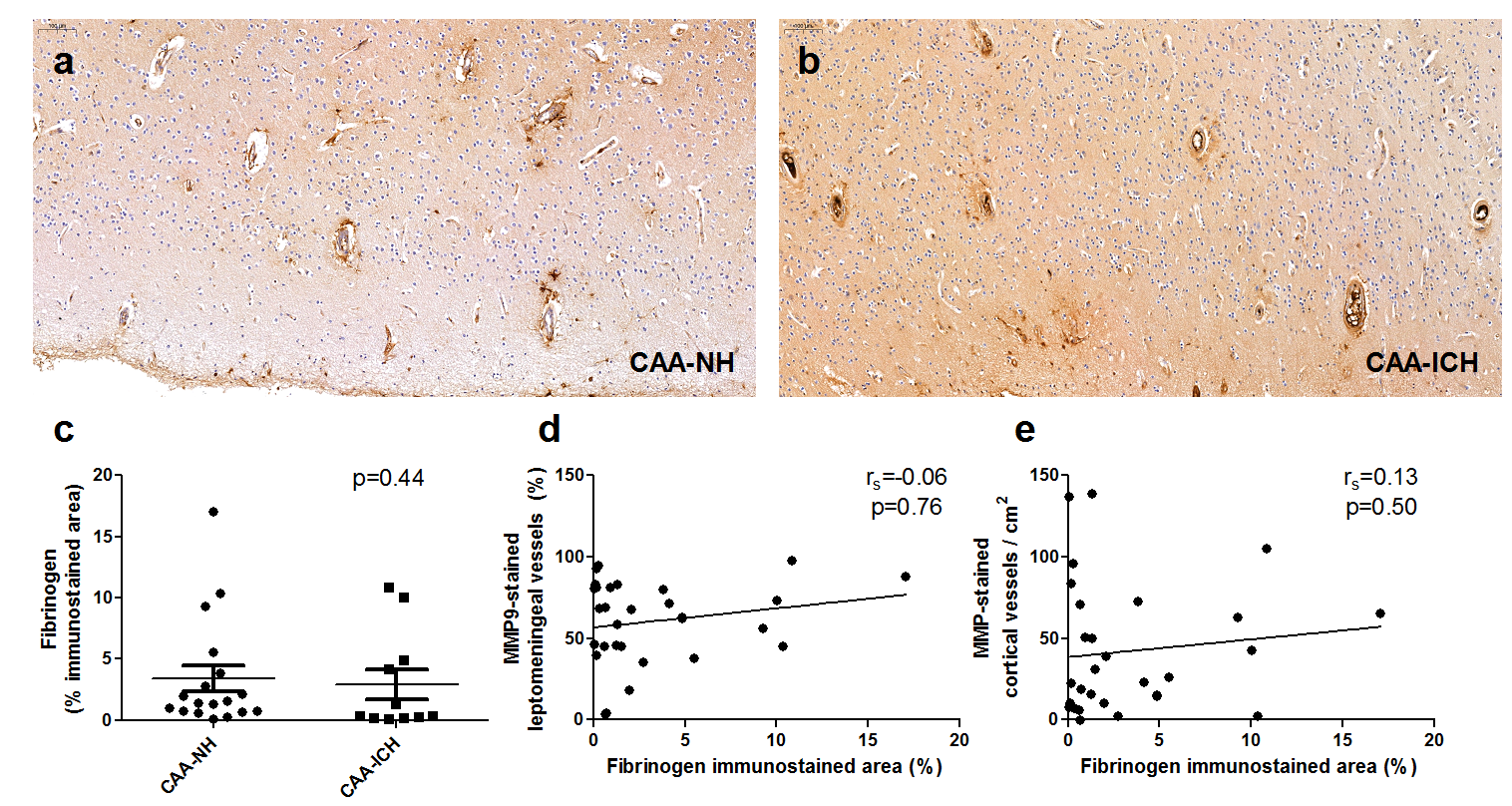


**Additional file 6.** Cortical fibrinogen staining in CAA-NH and CAA-ICH cases. Fibrinogen immunostaining, indicative of BBB leakage, was assessed in CAA-NH (a) and CAA-ICH (b) cases. The immunostained area did not differ between both groups (c). Furthermore, the percentages of MMP9-stained leptomeningeal vessels (d) and numbers of the MMP9-stained cortical vessels (e) did not correlate with fibrinogen staining. Scale bar = 100 µm. CAA-NH = CAA-non haemorrhagic, CAA-ICH = CAA-related ICH.

For fibrinogen antigen retrieval, sections were treated with 32x diluted proteinase K (Qiagen, 19133) for 5 minutes at RT. Sections were incubated 60 minutes at RT with mouse-anti-fibrinogen (A0080, DAK0). For quantification of fibrinogen staining, thirty regions of interest (ROIs) were selected in a zigzag sequence along the cortical ribbon. Using Fiji software (v 1.51), the percentage of positive pixels was determined, using a threshold to discriminate specific staining from background signal. The percentage immunostained area of every section was calculated by averaging the percentages of positive pixels of the thirty ROIs, and compared between groups using linear regression with age and sex as covariates. Spearman correlation analysis was used to assess the correlation between cortical and leptomeningeal MMP9 staining and fibrinogen immunostaining.
